# Regression-Based Classification of the Middle-Latency Auditory-Evoked Potentials in Vestibular Migraine and Concussion Patients with Dizziness

**DOI:** 10.3390/brainsci15010001

**Published:** 2024-12-24

**Authors:** Carolina Beppi, Daniel Agostino, Antonella Palla, Nina Feddermann-Demont, Julia Dlugaiczyk, Dominik Straumann

**Affiliations:** 1 Neuroscience Center Zurich, University and ETH Zurich, CH-8091 Zurich, Switzerland; dominik.straumann@usz.ch; 2 Interdisciplinary Center for Neurological and Vestibular Disorders, Department of Neurology, University Hospital Zurich, CH-8091 Zurich, Switzerland; daniel.agostino@usz.ch (D.A.); antpalla@gmail.com (A.P.); julia.dlugaiczyk@usz.ch (J.D.); 3 Clinical Neuroscience Center, University Hospital Zurich, CH-8091 Zurich, Switzerland; 4 Sports Neuroscience, University of Zurich, CH-8006 Zurich, Switzerland; nina.feddermann@braincare.swiss; 5 BrainCare Medical Group, CH-8002 Zurich, Switzerland; 6 Neurocenter Bellevue Medical Group, CH-8001 Zurich, Switzerland; 7 Department of ORL, University Hospital Zurich, CH-8091 Zurich, Switzerland

**Keywords:** habituation, auditory-evoked potentials, concussion, vestibular migraine, logistic regression, multiple regression, group classification

## Abstract

Background/Objectives: The auditory middle-latency responses (AMLRs) assess central sensory processing beyond the brainstem and serve as a measure of sensory gating. They have clinical relevance in the diagnosis of neurological conditions. In this study, magnitude and habituation of the AMLRs were tested for sensitivity and specificity in classifying dizzy patients with vestibular migraine (VM) and post-concussive syndrome. Methods: Twenty-three healthy individuals, 12 concussion and 26 VM patients were recruited. AMLR were recorded performing five blocks of 200 binaural click-stimulations at 60 dB sensation level with a repetition rate of 6.1 Hz. Reduction in P0, Na and Pa magnitudes between blocks was measured. Group classifications were performed through logistic and multiple regression. Results: Among healthy subjects, a consistent P0 and Na habituation can be observed. Concussed subjects show control-like Na habituation, despite a lower magnitude, while P0 habituation was negligible. VM patients showed poor habituation for all waves. Regression analyses suggest that P0 and Na better distinguish healthy subjects from neurological patients, whereas Pa best distinguishes concussion from VM patients. Conclusions: The results support that AMLR habituation can contribute to unraveling different mechanisms of dizziness due to concussion compared to VM, providing insights that can complement routine diagnostic assessments.

## 1. Introduction

The auditory-evoked potentials (AEPs) have an established use in assessing neural abnormalities along the hearing pathway [1]. They provide accurate measures of synchronous firing activity from the cochlea to the cortex [2] and are hence ideal candidates for identifying eventual functional aberrations along the hearing pathway in clinical neurophysiological groups. Objective audiometry, such as recording AEPs, complements the result of subjective hearing tests, e.g., pure-tone audiometry (PTA). In particular, AEPs aid in the diagnosis of auditory synaptopathy and neuronopathy (i.e., normal otoacoustic emissions (OAEs), but compromised auditory brainstem responses (ABRs) (which we will describe in the next paragraph) and the topodiagnosis of central auditory disorders. Moreover, ABRs are not significantly affected by sleep or anaesthesia and are, therefore, commonly used to determine hearing thresholds in patients unable to undergo subjective audiometry (e.g., infants and patients with cognitive disorders) [3,4,5].

The different AEP components are grouped into the early-latency, middle-latency, and late-latency subtypes, according to their peak latencies relative to the auditory stimulus [6]. The first AEP subtype is also known as the ABRs. These comprise of waves I, II, III, IV, V, VI, VII, all occurring within the first 10 ms post-stimulus, which originate from the auditory nerve and progress through the cochlear nucleus, terminating at the inferior colliculi. The second AEP subtype is known as auditory middle-latency responses (AMLR), and includes the N0 (8–10 ms), P0 (11–13 ms), Na (15–25 ms), Pa (24–35 ms), Nb (35–50 ms) and Pb/P1 (50–80 ms) components [4,7,8,9]. The AMLR amplitudes, particularly the Na–Pa wave, are larger than the ABR and range from 0.5 to 3.0 μV (mean: 1.0 μV) [7,8,10]. The final subtype—the auditory late-latency responses (ALLRs) or auditory event-related potentials (aERPs)—comprises of slower cortical waves arising after 100 ms. Several ALLR components have been defined, among which are the well-known P100 and P300 responses (reviewed in Beppi et al. [11]). The ALLR amplitudes are larger than the AMLR and range from 3 to 15 μV [8]. The AMLRs evaluate central auditory processing beyond the level of the brainstem, from the inferior colliculi through the thalamocortical tracts, connecting the thalamic medial geniculate nucleus to the lateral superior temporal gyrus [12,13]. Specifically, the neural generators of the earliest N0 and P0 components putatively have subcortical origin [14]. The Na–Pa complex instead might be the first primary cortical-level activity, at the level of the first transverse gyrus, in the postero-medial part [14].

In clinical practice, AMLR recording is more challenging compared to ABR and ALLR recording due to several reasons: (I) more generally, due to the lack of normative data and less established standardised protocols; (II) responses may be confused with artifacts of the posterior auricular muscle due to similar latencies; (III) amplitudes may be reduced by sleep and anaesthesia; (IV) the variability of AMLR, as compared to ABR responses, is higher as the source of the potentials is closer to the scalp surface; (V) AMLR cannot be reliably detected in children <10 years despite normal hearing, probably due to maturation issues of the central auditory pathways [4,15,16].

ABR and AMLR alterations are well-documented in migraine patients. The term “vestibular migraine” (VM) characterizes vestibular symptoms associated with migraine. The diagnostic criteria for VM were defined by the Bárány Society and the International Headache Society in 2012 and updated in 2022 [17]. In summary, the diagnostic criteria include the presence of at least five moderate-to-severe attacks of vestibular symptoms (5 min–72 h), a past/present history of migraine and at least one typical migraine feature in ≥50% of attacks (migraine-type headache, photo- and phonophobia, visual aura). VM patients report a reduced quality of life and are particularly prone to developing anxiety and depression compared to patients with other vestibular disorders [17,18,19].

There is evidence of delayed I, III, and V peak latencies in VM patients and delayed V peak latency in migraine patients, relative to healthy subjects [20]. The AMLR were also found altered in VM patients, in the form of a lack of habituation of Na–Pa amplitude [10], but less data are available for structurally more “widespread” neurophysiological disorders such as concussion. Concussion (CCS), or mild Traumatic Brain Injury (mTBI), is a transient neurological dysfunction resulting from biomechanical forces transmitted to the brain, commonly from direct blows or impulsive motions [21]. It is the most common form of TBI (75%), with an estimated annual incidence of 1–6/1000 affected individuals [22,23]. Clinical signs and symptoms include headache, dizziness, impaired concentration, sleep disorders, blurred vision, hypersensitivity to light or noise, imbalance, confusion, incoordination, nausea, and in some cases, transient loss of consciousness [24,25]. Upon impact, the brain undergoes metabolic shock characterized by ionic disruption and decreased energy disposal. Blood–brain barrier disruption may also occur [26]. Metabolic function usually restores within approximately 7 days, along with the resolution of acute symptoms. However, longer-lasting consequences may occur in repeated CCS, young adults, and children [27,28,29,30,31]. Unlike severe TBIs, CCS does not lead to visible focal injuries on standard brain-imaging methods, posing challenges to complete understanding of neuropathological events [32,33]. The diffuse functional (or metabolic) changes affecting symptomatic mild TBI patients with seemingly normal imaging [34,35] might affect AMLR amplitudes and habituation, in analogy to zebrafish results [36,37].

The AMLRs have shown amplitude and onset alterations in individuals that incurred a concussion [38,39], in patients with persistent postural perceptual dizziness (PPPD) [40,41], as well as in schizophrenic patients [42,43,44]. Subjects with VM also show impaired AMLR habituation [10]. While VM and CCS are two distinct neurological conditions, they often show overlapping symptoms, including headache and vestibular disturbances/deficits [45], and may co-occur in the same patient. Also, individuals with migraines who then undergo a concussion, are reported to experience a temporary worsening of migraine symptoms [46,47,48].

The AMLRs have been used as a measure of sensory gating [49,50], namely the ability to filter out irrelevant information at early stages, preventing it from undergoing higher-order (primary) processing. This ability was found to be compromised in subjects who incurred a TBI—irrespective of its severity [51]—with evidence of reduced and delayed Na and Pa amplitudes [38,52,53]. However, negative findings or clinically insignificant differences have also been reported [54,55]. The inconsistency might stem from variability in lesion level, experimental protocols and time interval between the injury and the test. This calls for the need of more standardised protocols in the assessment of post-concussive auditory dysfunctions. Habituation of the AMLRs has always been quantified as a Na–Pa reduction in magnitude over repeated blocks of stimulation (e.g., Murofushi et al. [12]). Here, in this work, we propose and explore new ways of assessing habituation, to compare the relative importance of different metrics. In addition to Na–Pa, we will account for the P0–Na component, and also for the *P0–Na–Pa complex*—a compound measure of the P0, Na, Pa peaks as a whole—and compare the relative utility of each measure.

This study aims to expand Murofushi et al.’s work [12], investigating sensory processing in dizzy patients with post-concussive syndrome and VM, as measured by the AMLR habituation and magnitude. Despite having distinct diagnostic criteria, deciding whether a clinical presentation is a sign of chronic CCS or pre-existing VM can be challenging, particularly when the clinical presentation is ambiguous. Investigating neurophysiological differences between VM and CCS might not only be informative in the diagnostics process, but also in the development of targeted therapies. This study will thus investigate if AMLRs can represent a helpful complementary tool aiding clinical decision-making when facing symptoms of an uncertain nature. AMLR magnitude and habituation will be computed and used to perform group classification among healthy control subjects (CNT), chronic CCS and VM patients. The study aims to capture any sensory processing alterations that may persist beyond the immediate effects of an acute VM attack, in comparison with the long-term effects of a single concussion—two conditions that in the acute phase often share symptomatic similarities. This approach will shed light on how these conditions may affect patients during their daily lives, and not just in proximity to an acute episode. We hypothesized that the groups would be efficiently classified based on their AMLR magnitude and habituation, and that the computed AMLR metrics would have different classificatory ability.

## 2. Materials and Methods

### 2.1. Ethical Approval

This study was approved by the Cantonal Ethics Committee of Zurich (BASEC-Nr. 2020-02853).

### 2.2. Participants

The participants included 12 CCS patients (age M = 36.27 years old, SD = 9.98) of which 5 were females. All subjects reported dizziness at the time of their admittance to the hospital. The patients underwent the AMLR assessment within 18 months after injury and not earlier than 2.5 months. They were, therefore, all chronic, according to the Concussion in Sport Group (CISG) international Consensus Statement definition of sport-related CCS [56] and the American Congress of Rehabilitation Medicine (ACRM) diagnostic criteria for mild traumatic brain injury [57]. The second patient group comprised 26 VM patients of whom 7 were females (age M = 40.23 years old, SD = 13.25). The VM patients were tested in the interictal period, meaning no participants were tested during or immediately after a migraine attack, to capture the chronic sensory processing characteristics that may persist beyond the acute symptomatic phase. The time since the last migraine attack varied among VM participants, ranging from a few days to several months. The CNT group included 23 individuals of which 13 females (age M = 30.26, SD = 7.50). Specific inclusion and exclusion criteria for the patient groups are listed and described below.

#### 2.2.1. Inclusion Criteria

##### CCS Group

1. Any patient aged 18–65 years old with a confirmed diagnosis of a single concussion, at least 3 months prior to the test, fulfilling the CISG definition of sport-related CCS [56] and the ACRM diagnostic criteria for mild TBI [57]. Accordingly, a mild TBI is defined as a traumatically induced physiological disruption of brain function, evidenced by: (I) at least one clinical sign attributed to the attributable to brain injury or having two (or more) symptoms and one (or more) abnormal clinical examination or laboratory findings; (II) loss of consciousness with maximal duration of 30 min, a Glasgow Coma Scale between 13 and 15 and a post-traumatic amnesia with maximal duration of 24 h; (IV) no evidence of any abnormalities on neuroimaging.
2. Cerebral MRI including susceptibility-weighted imaging confirming no signs of brain trauma.
3. Normal hearing in the PTA—defined as ≤20 dB nHL for frequencies between 500 and 4000 Hz—and hearing threshold ≤10 dB for click stimuli in both ears. This measure was taken in order to exclude any heterogeneity in hearing function amongst the study groups.

##### VM Group

1. Any patient aged 18–65 years old diagnosed with VM in accordance with the Bárány Society [17].
2. Normal hearing in the PTA—defined as ≤20 dB nHL for frequencies between 500 and 4000 Hz—and hearing threshold ≤10 dB for click stimuli in both ears. This measure was taken in order to exclude any heterogeneity in hearing function amongst the study groups.

#### 2.2.2. Exclusion Criteria

##### CCS Group

1. Presence of neurological disorders other than concussion (besides primary headache disorders), central vestibular disorders, ocular disorders, or/and psychiatric (excl. ADHD) disorders. Central vestibular and psychiatric disorders were excluded by (I) taking the patients’ history with special regard to those disorders and respective medication, (II) performing a detailed clinical neurological examination in all patients and (III) studying the patients’ records including brain imaging, when available.
2. Patients with acute concussion (<3 months since injury).
3. Patients who incurred a CCS that have or did experience one (or more) of the following: (A) a post-traumatic loss of consciousness with a duration exceeding 30 min; (B) a Glasgow Coma Scale below 13; (C) a post-traumatic amnesia lasting more than 24 h; (D) post-traumatic evidence of abnormalities on neuroimaging.
4. History of migraine or VM to ensure that the neurophysiological differences observed were attributable selectively to the condition under study.
5. History of multiple CCS to reduce data variability and a more homogenous study population, as neurophysiological responses after repeated CCS could differ significantly from those observed after a single CCS.

##### VM Group

1. History of CCS to ensure that the neurophysiological differences observed were attributable selectively to the condition under study.
2. Presence of hearing and balance disorders due to other causes. In order to exclude hearing disorders due to other causes, study subjects underwent an otological examination (including otoscopy, Weber, Rinne and Valsalva test) by an ENT specialist and a PTA. In addition, audiological history was taken and patient records including audiological examinations were reviewed. Other vestibular disorders were excluded by patients’ history, clinical neurotological examination and vestibular testing as indicated by the clinical phenotype.
3. Subjects who had an attack 1 or 2 days prior to the AMLR test.

### 2.3. Recording and Stimulatory Parameters

AMLR were recorded using the module “EP25-Clinical AEP” of the Eclipse EP Software and the EP hardware platform by Interacoustics (Eden Prairie, Minnesota, United States). The selected electrodes were four “Ambu BlueSensor N” by Ambu A/S (Ballerup, Denmark). The ground electrode was located on the low forehead (FPz). The active electrode was placed on the vertex (Cz), whereas the inactive electrodes were placed on the mastoids. The testing protocol was adapted from Murofushi et al. [12]. It consisted of five blocks with 200 trials. Click stimulation (0.1 ms) was set to 60 dB sensation level, which is an intensity level shown to favour Na–Pa habituation [14]. Clicks were binaurally presented through headphones (model DD45, 0.01 kΩ, Interacoustics), with a repetition rate of 6.1 Hz. Signals were bandpass filtered (3.3–1500 Hz) and a total of 1000 responses were averaged and divided into 5 blocks of 200 trials each.

### 2.4. Procedure

After reading the information sheet, the subjects who signed their informed consent to take part in the study were recruited. The subjects were seated comfortably on a chair positioned 1.5 m in front of a wall. Electrodes and headphones were then applied. The experiment began only when an acceptable impedance (<3 Ω) could be obtained and was consistent (SD of Ω < 1.5) across all channels. The subjects were thereafter instructed to fixate a blue sticker on the wall at the eye level for the whole duration of the experiment (<5 min) and asked to avoid blinking and/or moving their body to reduce the incurrence of recording artefacts.

### 2.5. AMLR Labeling Nomenclature and Corrections

**Figure 1:**
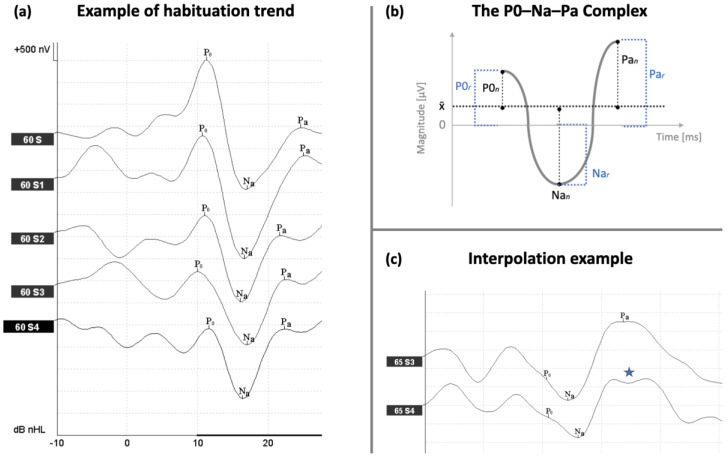
AMLR recording and computation. (**a**) Example of P0–Na–Pa waves labelling and habituation along repeated blocks (60S → 60S4). (**b**) Depiction of the three peaks composing the P0–Na–Pa complex: raw and baseline adjusted P0 (P0r and P0n, respectively), raw and baseline adjusted Na (Nar and Nan, respectively), raw and baseline-adjusted Pa (Par and Pan, respectively). The mean (per subject) equals the average magnitude of P0, Na and Pa. (**c**) Interpolation effect example. The “effect” is indicated with a blue star.

The nomenclature was adapted from Picton et al. [9]. Waves P0, Na, and Pa were identified as the reference positive and negative markers. P0 was defined as the first upward (normally positive) deflection within 20 ms post-stimulus. When no such deflections were identifiable, P0 was not labelled. Na was defined as the first downward (normally negative) deflection following P0, within 25 ms post-stimulus. Pa was defined as the first upward (normally positive) deflection following Na, within 35 ms post-stimulus. An example of wave labeling is provided in Figure 1. A correction was applied in cases where one component (P0, Na or Pa) would not appear in the expected timeframe, and instead two “unmerged” peaks—of an earlier and later latency, respectively—would occur (Figure 1). We refer to this event as *interpolation* effect. In such cases, the mean latency and mean amplitude of the two peaks would be computed and used as “corrected” values.

### 2.6. AMLR Measures

The raw P0, Na, and Pa amplitudes as well as the raw peak-to-peak P0–Na and Na–Pa amplitudes were measured for each block (1–5) and for each subject of all three groups. Additionally, the P0–Na–Pa complex (cmpx) in Figure 1, was computed as the absolute sum of the P0, Na and Pa amplitudes:

(1) $$cmpx=\left|P0\right|+\left|Na\right|+|Pa|$$

The raw P0, Na, and Pa magnitudes were then baseline-adjusted by subject, subtracting from the magnitude of each raw peak (P0, Na and Pa) the mean magnitude of the P0, Na and Pa raw peaks, which we will refer to as $\bar{x}$ (Figure 1). This method was chosen over standard normalisation methods (e.g., Z-score, min/max, decimal scaling) in that it reduced the impact of outliers and inter-subject variability, achieving an approximately normal distribution of data, thus making the data more amenable to statistical analyses and the linear regression model we employed, without requiring transformations, and hence introducing a potential risk of obscuring the real relationships we aimed to analyse. The *amplitude* of each measure was defined as the magnitude of each measure in block 1. The *offset* of each measure was defined as the mean magnitude of each measure in blocks 4 and 5. The *habituation* of each measure was defined as the delta (Δ) magnitude between its amplitude and offset.

### 2.7. Statistical Analyses

Normality of data-distribution for parametric tests was assessed by checking: (I) the presence/absence of any significant outliers; (II) whether the skewness and kurtosis were close to 0; (III) the significance of the Shapiro–Wilk test (IV) whether data histograms were bell-shaped; (V) whether the Q-Q plots were an approximately a straight line. An independent-measures 1-way ANOVA was conducted to test for between-group differences in age (1st ANOVA). A clinically significant effect of hearing threshold would be assumed only if >10 dB (e.g., Carl et al. [58]). Since the click hearing threshold of all participants ranged between 5 and 10 dB nHL, no statistical tests to assess the presence of any significant differences across groups were performed. The percentage of positive habituation and the magnitude reduction were calculated for all groups and all measures. Positive or effective habituation was defined as a reduction in magnitude between block 1 and the mean of blocks 4 and 5. This means if a subject’s P0 magnitude in block 1 was equal to or lower than the mean magnitude in blocks 4 and 5, this would not be accounted as effective habituation. The choice to compare block 1 with the mean of blocks 4 and 5, instead of block 5, aimed at mitigating eventual within-subject magnitude fluctuations at the final 2 blocks of the test, which may reflect fatigue and attentional shifts. An average response measure of blocks 4 and 5 was thus computed to mitigate any unexplained variability that might occur during this later phase, thus obtaining a more robust estimation of individual responses in the later stages of the test.

Non-parametric Kruskal–Wallis ANOVAs were run to assess for between-group differences in habituation, for each variable (baseline-adjusted P0, Na, Pa as well as peak-to-peak P0–Na and Na–Pa). A receiver operating characteristic (ROC) curve and its respective area under the curve (AUC) for 3-class classification by logistic regression was produced for each measure, using the inbuilt MATLAB function “perfcurve”. A combination of the amplitude (magnitude in block 1) and habituation (delta magnitude between block 1 and block 5) were used as input values. Multiple logistic regression was used to explore which combination of measures could provide the highest classification accuracy. Support vector machine (SVM) was used to rank the measures imputation based on the output weights. Variables were fitted using a logistic binomial model (MATLAB function “fitglm”).

For all statistical analyses performed, a minimal significance level of 0.05 was always assumed. All analyses and statistics were conducted using MATLAB 2021b (The MathWorks Inc., Natick, MA, USA) and SPSS Statistics version 27.0 (IBM Corp., Armonk, NY, USA).

## 3. Results

### 3.1. Age

The assumptions of normality—as assessed using histograms and Q-Q plots—for the age distributions for all groups were found tenable. The age variable failed the homogeneity of variance assumption, with F(2,57) = 4.01, *p* = 0.023). Welch statistics have thus been chosen to evaluate the effect of age. Games–Howell post hoc comparisons indicated that age of the CNT group (M = 30.26, SD = 7.5) was significantly lower than that of the VM group (M = 40.23, SD = 13.25) with *p* = 0.006. No other significant differences were found.

### 3.2. AMLR Habituation

**Figure 2:**
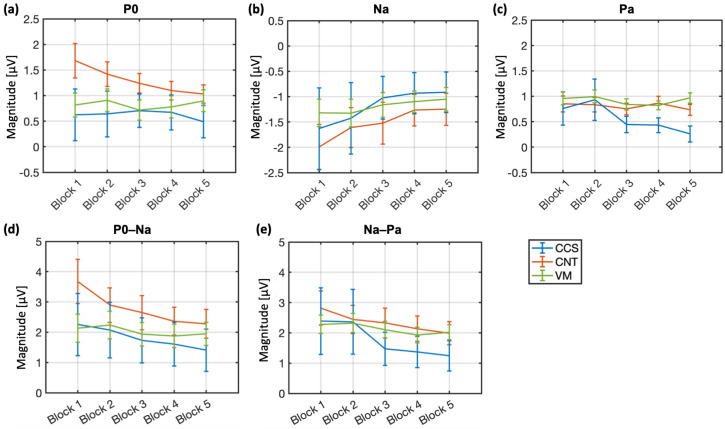
AMLR habituation over the blocks. (**a**–**c**) Change in mean (+/− 1 SE) baseline-adjusted P0, Na and Pa amplitudes across blocks of all groups. (**d**,**e**): change in mean (+/− 1 SE) peak-to-peak P0–Na and Na–Pa amplitudes across the 5 blocks (200 trials each) for all groups: concussion (CCS), control (CNT) and vestibular migraine (VM).

Among CNT subjects, a consistent P0 and Na habituation can be observed (Figure 2). CCS patients show a similar Na habituation function as CNT subjects, despite a lower amplitude and offset. However, as opposed to CNT subjects, they display an effective Pa habituation but a null P0 habituation. Patients with VM showed poor habituation for all waves. The peak-to-peak P0–Na and Na–Pa waves show a similar habituation pattern across blocks and groups as Na. The CCS group habituates in a similar fashion and proportion as the CNT group—despite having a lower amplitude (magnitude of block 1). The VM group shows little-to-null habituation (Figure 2).

**Figure 3:**
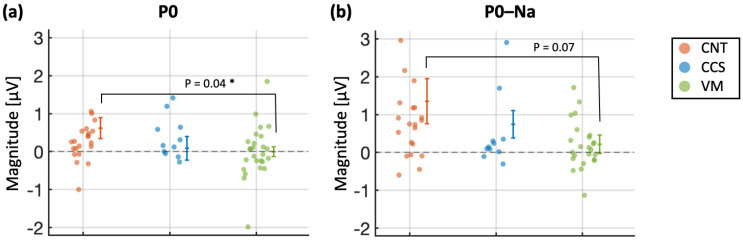
Statistically significant between-group differences in AMLR habituation. (**a**,**b**) P0 and P0–Na habituation for all subjects (dots) and all groups: control (CNT), concussion (CCS), vestibular migraine (VM). Effective habituation (i.e., subjects whose peak’s magnitude in block 1 was larger than the peak’s magnitude in the last two blocks) is indicated by cases falling above the 0 line (i.e., no difference between blocks). Cases showing no habituation would fall below the 0 line. Statistically significant differences, as well mean and standard error, are indicated with asterisks, with * *p* < 0.05.

Individual differences in habituation, as assessed using Kruskal–Wallis ANOVAs, that were statistically significant at the group level are provided in Figure 3. On a quantitative level, P0 and P0–Na were the two measures showing the highest proportion of effective habituation in CNT subjects (78% and 74%, respectively, with a magnitude reduction of 56–62%), while only 50% of the VM subjects habituated. Na showed an approximately equal percentage of positive habituation in the CNT and CCS groups (78% and 68%, respectively) with a magnitude reduction of about 60% for both. Whereas the proportion of effective Na–Pa and Pa habituation was highest for the CCS patients (67%) against the 43% and 53% of the CNT and VM groups, respectively.

### 3.3. ROC Curves for 1-by-2 Classification Using Logistic Regression

**Figure 4:**
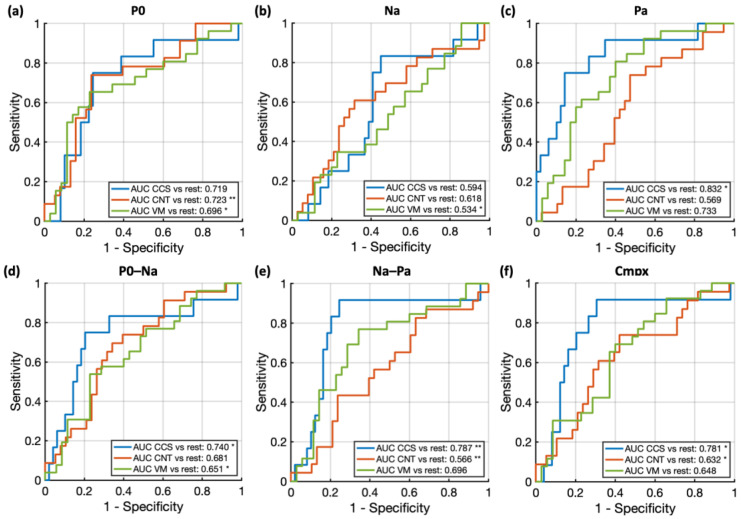
1-by-2 group classifications by logistic regression. (**a**–**c**) ROC curves illustrating the model’s performance for classifying the three groups (vestibular migraine (VM), concussion (CCS) and healthy controls (CNT)) in 1-by-2 comparisons, using each individual baseline-adjusted measure (from left to right: P0, Na, Pa). (**d**–**f**) ROC curves for the model’s classificatory performance using raw peak-to-peak measures (P0–Na, Na–Pa) and the combined P0–Na–Pa complex, showing the classification accuracy based on these raw measures for distinguishing the three groups. Statistically significant *p*-values (i.e., AUC significantly different from 0.05) for each AUC based on the Hanley and McNeil method are indicated with asterisks, with * *p* < 0.05, ** *p* < 0.01. Non-significant *p*-values are not shown.

The ROC curves in Figure 4 highlight that among the baseline-adjusted peaks:

1. P0 reaches the highest AUC when distinguishing the CNT group from the rest and was, on a general level, the measure achieving the highest AUCs for all 1-by-2 group comparisons. Statistically significant AUCs for the P0 measure were achieved for the following comparisons: CNT vs. rest (AUC = 0.723, *p* = 0.0065) and VM vs. rest (AUC = 0.696, *p* = 0.0118). For the Po–Na measure, statistically significant AUC were instead achieved for the CCS vs. rest comparison (AUC = 0.740, *p* = 0.04) and for the VM vs. rest comparison (AUC = 0.651, *p* = 0.05).
2. Na was instead the most “disadvantaged” measure, achieving the lowest AUCs. A statistically significant AUC for the Na measure was achieved for the VM vs. rest comparison only (AUC = 0.534, *p* = 0.045).
3. Pa and Na–Pa prevailed in the distinction of CCS patients from the rest. For the Pa measure, only the CCS vs. rest comparison approached statistical significance (AUC = 0.832, *p* = 0.0007). Statistically significant AUCs for the Na–Pa measure were achieved for the CCS vs. rest comparison (AUC = 0.787, *p* = 0.0065) and for the CNT vs. rest comparison (AUC = 0.566, *p* = 0.0071).
4. Cmpx showed a balanced class-prediction ability across 1-by-2 group comparisons. Statistically significant AUCs for the cmpx measure included: CCS vs. rest (AUC = 0.781, *p* = 0.04); CNT vs. rest (AUC = 0.632, *p* = 0.0261).

### 3.4. ROC Curves for 1-by-1 Classification Using Multiple Regression

**Figure 5:**
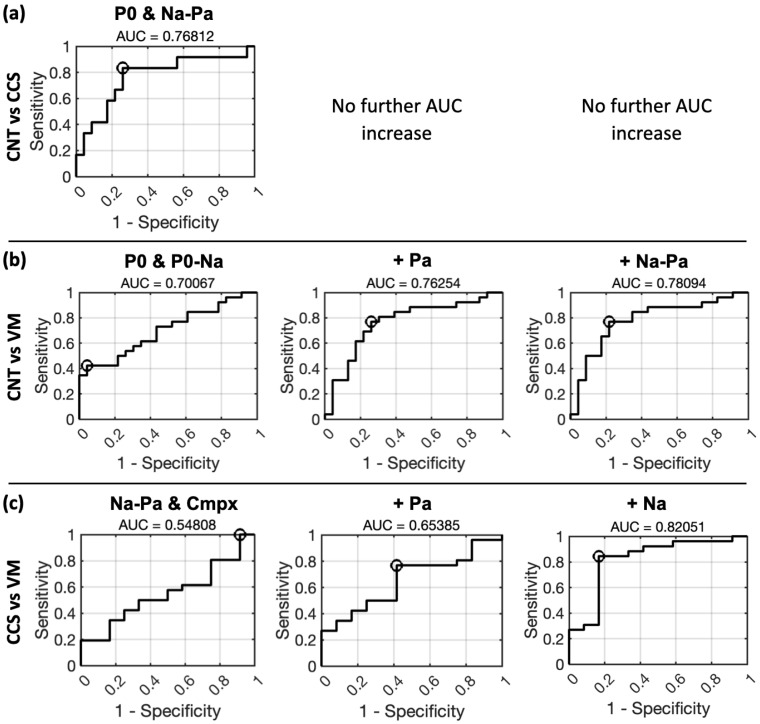
1-by-1 group classifications by multiple regression. ROC curves for 1-by-1 classification using multiple egression based on the absolute amplitude (i.e., peak magnitudes in block 1) and habituation of each metric in a stepwise fashion. The comparisons are as follows: (**a**) healthy control (CNT) vs. concussion (CCS) in the top panel, (**b**) CNT vs. vestibular migraine (VM) in the middle panel, and (**c**) CCS vs. VM in the bottom panel. The black circle indicates the optimal operating point (OOP) for each comparison.

Next, we explored which combination of metrics (i.e., P0, Na, Pa, P0–Na, Na–Pa, cmpx) grants the best classification accuracy in 1-by-1 group comparisons, through a multiple regression approach. The model was trained on each peak’s amplitude (i.e., magnitude in block 1) and habituation. The results indicate that P0 is the first-best measure for distinguishing CNT subjects from CCS or VM patients, while Na and Pa prevail when distinguishing between CCS and VM patients (Figure 5).

## 4. Discussion

Recently, Murofushi et al. [12] proposed a simple protocol for the recording and assessment of AMLR habituation, reporting lack of habituation—defined as a response decrement as a result of repeated stimulation—in patients with VM, a neurological dysfunction that shares several symptoms with mTBI, including headache and vestibular disturbances [39]. Our study aimed at designing a more comprehensive protocol for the study of AMLR habituation, based on Murofushi et al. [12]. We assessed two neurological groups—chronic CCS and VM patients in the interictal period—for their AMLR amplitude, and habituation in comparison with CNT subjects, to understand whether these measures could be of diagnostic utility. We evaluated the relative class-predicting ability of different AMLR measures, using both standard metrics (i.e., Na–Pa) and novel metrics such as baseline-adjusted P0, Na and Pa, as well as the P0–Na and the P0–Na–Pa complex. We used logistic and multiple regressions to assess whether the experimental groups could be correctly classified based on their AMLRs.

The P0, Na, Pa, P0–Na, Na–Pa metrics were first quantified for their habituation pattern (along blocks) and proportion across the different clinical groups. On a gross level, the results indicate that the groups have different habituation trends along the blocks and can be distinguished based on their AMLRs. Different measures also show different habituation patterns, highlighting that the Po and Na components are susceptible to habituation, whilst Pa is not. In CNT subjects, P0, Na, P0–Na and Na–Pa amplitudes habituated with a prominent reduction, although not in all cases. This implies that habituation does not always or necessarily occur even in healthy subjects. This might be the case of subclinical VM patients, namely patients with a genetic makeup that could lead to clinical migraine, if environmental factors favor it. In the CNT group, Pa amplitude was generally the most stable AMLR component, showing relative stability along blocks. Consistently, Pa was previously reported as being the most robust AMLR component across subjects [59,60,61]. Taken overall, the robustness of Pa amplitude suggests that this component might reflect more basic aspects of sensory processing, such as the encoding of morphological features of a sensory stimulus, rather than its significance or relevance. This might explain its resistance to habituation in CNT subjects. VM patients show clear deficits of habituation, with a generally low proportion and magnitude of habituation for all the analysed measures (i.e., P0, Na, Pa, P0–Na, Na–Pa), consistently with Murofushi et al.’s findings [12]. CCS subjects showed on average control-like proportion and magnitude of habituation with Na, Po–Na and Na–Pa waves. This suggests that despite displaying a lower amplitude across all the blocks, the “habituation function” was preserved. In addition, a limited P0 habituation and a stronger Pa habituation were observed compared to CNT subjects. These findings indicate that, as tested by the AMLRs, chronic CCS patients display changes—rather than deficits—in sensory processing and integration, supporting past findings [38,39,52,53]. This might suggest that neural plasticity processes of functional reorganization might have acted to restore mechanisms of sensory processing and integration. Interestingly, recent studies suggest that individuals with persistent post-concussive symptoms display abnormal/deficient brain activations [62,63], while individuals recovering from post-concussive syndrome show frontal hyperactivations that are compensatory for neurocognitive/functional restoration [64,65].

For future research, it would be valuable to examine the patterns immediately after an injury and later in the same individual. This could provide significant insights into functional recovery. Additionally, one might consider separating CCS individuals into an early and late subgroup, based on the time that has passed since their injury. Such distinction would allow for testing whether the late group shows a functional recovery relative to the early subgroup, as highlighted by eventual changes in AMLR patterns and in the dynamic coordination of brain activity. In this context, hybrid methods—such as brain MRI-informed EEG—with source localisation technologies (e.g., Chang et al. [66]), could be considered to investigate whether the activation of frontal/executive structures reduces over time upon the approach of habituation (i.e., cessation or reduction of response). Such findings would be informative as to the frontal contribution to response habituation. One would in turn be able to assess whether CCS and VM patients show altered (control-relative) patterns. One might furthermore consider distinguishing VM and mTBI patients based on their imaging (positive or negative) to assess whether the AMLR assessments can capture functional alterations in CCS individuals beyond an apparently negative MRI or CT detections [29,30]. This would be indicative of the AMLR functional sensitivity to the neurological disorder.

Similarly, the variability in the time since the last attack for VM patients is a limitation of our study, in that sensory processing shortly after an attack compared to individuals who have not had an episode for an extended period might differ. We, therefore, suggest that future studies would also consider the time since the last migraine attack as a variable to control, or to divide VM patients into different segments, to better understand the acute and chronic effects of VM on sensory processing and AMLR habituation.

At the class-predictive level, when looking at single measures in 1-by-2 comparisons, Po seems to be the best of the P0–Na–Pa complex components at distinguishing CNT subjects from the two patient groups (Figure 4, top panel), while Pa and Na–Pa showed an advantage in distinguishing CCS from VM and CNT. Na was instead the measure with the lowest classificatory ability for all 1-by-2 comparisons, achieving the lowest AUCs. Cmpx showed a balanced class-prediction ability across 1-by-2 group comparisons. Taken together, the findings suggest that the classificatory ability of each measure might vary consistently based on the comparison groups, and that grouping different conditions together with “1 vs. rest” approaches might not be the most suited approach to understand the relative significance or weight of our measures.

We thus proceeded by comparing the measures for their classificatory ability in a stepwise fashion (i.e., multiple regression) in 1-by-1 group comparisons. The results once again confirm that P0 is the best at distinguishing CNT subjects from CCS (Figure 5a) and VM patients (Figure 5b). This is not surprising, considering that P0 is a measure that habituates in CNT subjects, but not in CCS and VM subjects. For a similar reason, Na–Pa—habituating in CCS more than CNT, and not habituating at all for VM patients—also appears to have an important “weight”, contributing as the second (Figure 5a) and first (Figure 5c) best class-predicting variable. When comparing VM and CCS patients, P0 unsurprisingly loses “weight” relative to Pa and Na (Figure 5c). This is likely related to the fact that both groups show no habituation (i.e., a stable amplitude) to this component. In the VM–CCS comparison, Na, Pa and Na–Pa instead become better predictors. This is consistent with the fact that both Na and Pa mildly habituate in CCS but not VM. However, it is important to note that case history is also useful for distinguishing between VM and CCS. Cmpx showed a good classificatory ability when distinguishing concussion and VM patients (Figure 5b). It can thus be regarded as a helpful summary measure, and its use might be considered when in need of a quick screening measure to inform the making of differential diagnosis.

Taken overall, our results evidence distinct AMLR patterns among CNT subjects, VM and CCS patients, suggesting that the underlying pathophysiological processes might be different. This would explain why migraine treatments might not yield the same benefits for post-concussion syndrome. Post-concussive symptoms should hence not be viewed as a first-time migraine manifestation, and more targeted, specialised, interventions would be necessary to address the pathophysiology of post-concussive symptoms and improve clinical outcomes. CCS patients should be treated by individually tailored multimodal interventions including pharmacology—in accordance with current guidelines—and combined graded physical exercise, vestibular rehabilitation, manual neck therapy and psychological treatment [67,68,69]. While VM and CCS may co-occur, and post-concussion symptomatology (e.g., headaches and vertigo) shares phenomenological similarities with VM, the identification of AMLR patterns uniquely associated with each condition might be valuable in clinical cases of diagnostic unclarity. Although the results of the regression-based investigation are significant and contribute to knowledge, the AUC values do not achieve sufficient sensitivity for use as a standalone diagnostic tool but should rather be used as an additional neurological screening tool to be used in combination with standard tests used in clinical practice. Particularly, for cases presenting overlapping symptoms and unclear history, AMLR testing as a complementary tool, could aid in the identification of the more dominant underlying pathology. As such, the identification of different neurophysiological AMLR patterns could contribute to the definition of more targeted therapies.

### Further Limitations, Challenges and Future Directions

Some important limitations in our study must be acknowledged. First, the limited number and non-homogeneous distribution of subjects, and particularly, the higher number of VM (N = 26) and CNT (N = 23) subjects compared to the CCS group (N = 12 patients). Second, age showed a significant effect between the VM and CNT groups. Considering such limitations, it is important to take the results and interpretations of this study with caution. Thus, while our findings indicate different AMLR patterns between VM and CCS patients, the data are preliminary/premature, and definitive conclusions about the diagnostic specificity of these measures cannot yet be drawn. Appropriate data corrections, which would control for such an effect, would be needed for an unbiased interpretation of the findings. While our sample sizes did not allow such corrections, for future follow-up studies, we recommend the selection of a better age-matched CNT group, or at least the collection of larger datasets, enabling retrospective data adjustments factoring out eventual age variabilities between groups, hence ensuring unbiased results and univocal conclusions. Studies collecting larger samples would be required to confirm and extend our findings, hence validating the diagnostic potential of the AMLRs in distinguishing VM from chronic CCS, and other neurological conditions.

Importantly, migraineurs with a history of CCS were not included in our study. Follow-up research might consider including patients with comorbid VM and CCS to investigate their interaction and treatments that could potentially benefit this subgroup. Studies focusing on disentangling the biochemical cascade and functional differences between post-concussive syndrome and migraine would provide relevant insights feeding into the development of tailored treatments. Also, including acute and chronic CCS subgroups could provide relevant insights into the AMLR investigation, in comparison with the VM group. We aim to follow up with the next study, precisely focusing on this matter. Future studies should not only aim to replicate these findings but also explore additional biomarkers that could complement AMLRs in clinical practice.

It is important to highlight that while our study focused on VM and CCS, similar abnormalities in AMLR habituation could be present in other neurological (and non-neurological) conditions, such as essential tremor [70], multiple sclerosis [71], PPPD [41], and learning disabilities [72]. Co-occurrence of VM and PPPD is observed in clinical practice: It is estimated that around 25% of patients with VM develop PPPD [73]. In this context, AMLRs might serve as one tool to decipher possible common pathophysiological factors in these two disorders. Comparative studies assessing the relative sensitivity and specificity of the AMLR magnitude and habituation across a broader range of neurological conditions in larger samples remain unexplored. Further research is needed to determine specific AMLR signature alternations that might be specific to specific neurological subgroups that are yet to be validated. It should also be considered that AMLR measures might be more sensitive to a specific symptom rather than a given diagnosis. An alternative approach to analysis would be to group subjects based on symptoms instead of their (suspected) diagnosis. Headache, nausea, hypersensitivity, and vestibular symptoms including (typically non-spinning) vertigo, dizziness and unsteadiness—often triggered or exacerbated by active or passive motion, physical effort and/or relative motion of the visual surrounding—are common in the conditions of mild TBI [57] and VM [45] and may not be ideal for distinguishing features. Confusion, impaired concentration, and foggy/sluggish feeling are more specific symptoms of mTBI [74], while a moderate–severe frontal bilateral throbbing/pulsing head pain (often associated with photo- and phono-phobia and triggered by physical/cognitive effort and sleep deprivation), a more specific symptom of migraine [75]. A comprehensive description of the migraine phenotype is detailed by Ashina et al. [76] and is based on the International Classification of Headache Disorders (ICHD-3) [77]. These symptoms are hence candidates to act as distinguishing features of the different clinical groups. One would finally assess the AMLR measures’ specificity for the same distinguishing clinical symptoms. It is important to mention that examining the evoked potentials in the vestibular (e.g., Suleiman et al. [78]), somatosensory (e.g., Desjardins et al. [79]; Zumsteg et al. [80]) and visual [81] modalities would be beneficial in offering a comprehensive neurological profile for concussion and migraine patients.

A further methodological challenge we encountered relates to selecting suited data normalisation methods. As described in our methods, we opted for a baseline adjustment procedure that practically consisted of subtracting the mean amplitude of the three peaks from each peak. This method was effective in reducing the impact of outliers and inter-subject variability, improving data normality and suitability to statistical analyses and linear regression. However, such an approach may introduce artifacts, such as translating selective alterations in a single peak onto other peaks. Depending on the data collected, researchers may find that standard normalisation methods such as min–max scaling, log scaling, Z-score, or Box–Cox normalizations may be more effective in their given dataset. Methods requiring data transformation might also introduce a potential risk of obscuring the real relationships between peaks. We, therefore, recommend that in future studies, different baseline adjustment and standard normalisation procedures are weighted out against each other for their specific benefits and risks to minimize potential bias while preserving data quality.

## 5. Conclusions

The results generally support the utility of AEPs as an informative complementary assessment tool to help define the clinical profile of VM and chronic CCS. Two main findings and practical clinical implications can be drawn from this study:

1. The results expand the work by Murofushi et al. [12], comparing the performance of VM patients with a different neurological group (i.e., chronic CCS) with related clinical symptoms, providing commonalities and distinguishing AMLR features. Deficits of AMLR habituation are evident in VM patients, whereas CCS patients display alterations—rather than deficits—in sensory processing and integration, possibly due to plasticity processes of functional reorganization that warrant further investigation. Particularly, for cases presenting overlapping symptoms and unclear history, the identification of different neurophysiological patterns could aid the identification of the more dominant underlying pathology. As such, AMLR testing as a complementary tool could contribute to the definition of more targeted therapies.
2. AMLR habituation is a helpful quantitative and qualitative measure to interpret and support the regression-based accuracy of group classifications. No AMLR measure can, in general, be thought of as an “absolute best” at distinguishing clinical groups. Each measure shows a relevant advantage depending on the comparison group: P0 for distinguishing CNT and CCS, P0 and P0–Na for distinguishing CNT and VM and finally Na and Pa for distinguishing CCS and VM.

Limitations and proposals for further investigation:

1. Despite distinct AMLR patterns having been identified for the VM, chronic concussion and healthy subjects, our results are still premature, and univocal inferences the diagnostic specificity of these measures cannot yet be derived.
2. The collection of larger datasets is required to improve statistical power and predictive ability and to control for possibly confounding factors like age.
3. Our methodological challenges point to the need for weighting different baseline adjustment and standard normalisation procedures against each other, considering their relative benefits and risks, to minimize potential bias while preserving data quality.
4. The inclusion of the acute concussion subgroup—(A) acute (<3 months since injury) and (B) chronic (>3 months) stage—would provide relevant insights into the AMLR investigation, in comparison with the VM group.
5. Follow-up studies might consider including patients with comorbid VM and CCS to explore their interaction and potentially beneficial treatments for this clinical subgroup.
6. The AMLR assessment could be extended to other neurological and psychiatric disorders sharing aspects of the clinical profile of CCS patients, such as long COVID-19 [82] and cognitive/neurological deficits related to dementia, multiple sclerosis and/or Parkinson’s disease.
7. It might also be relevant to try distinguishing groups based on the presence of given symptoms, instead of based on diagnosis. For example, headache and vestibular signs are common in mild TBI and (vestibular) migraine patients [39].
8. Future research should not only aim to replicate these findings but rather investigate additional biomarkers that might complement AMLR testing in clinical practice.
9. Cell recordings during repeated auditory stimuli could be considered to test whether a peripheral component of sensory adaptation contributes to response habituation. Whether sensory adaptation and central habituation are mutually exclusive processes is in fact still an open research question, and a matter of debate in the existing animal and human literature [83,84,85,86].
10. The assessment of evoked potentials in other sensory modalities—vestibular (e.g., Suleiman et al. [78]), somatosensory (e.g., Desjardins et al. [79]; Zumsteg et al. [80]), visual [81]—might be considered to further explore the neurological profile of CCS subjects in comparison with VM patients.

## Data Availability

The raw data supporting the conclusions of this article are available upon request from the corresponding author due to ethical reasons.
